# Acute and early HIV infection screening among men who have sex with men, a systematic review and meta‐analysis

**DOI:** 10.1002/jia2.25590

**Published:** 2020-10-01

**Authors:** Shaun Palmer, Maartje Dijkstra, Johannes CF Ket, Elizabeth W Wahome, Jeffrey Walimbwa, Evanson Gichuru, Elise M van der Elst, Maarten F Schim van der Loeff, Godelieve J de Bree, Eduard J Sanders

**Affiliations:** ^1^ Centre for Geographic Medicine Research – Coast Kenya Medical Research Institute Kilifi Kenya; ^2^ International AIDS Vaccine Initiative Amsterdam the Netherlands; ^3^ Department of Infectious Diseases Public Health Service Amsterdam Amsterdam the Netherlands; ^4^ Department of Internal Medicine Division of Infectious Diseases, and Amsterdam Institute for Infection and Immunity (AI&II) Amsterdam UMC University of Amsterdam Amsterdam the Netherlands; ^5^ Medical Library Vrije Universiteit Amsterdam Amsterdam the Netherlands; ^6^ ISHTAR MSM Health and Social Wellbeing Nairobi Kenya; ^7^ Department of Global Health, and Amsterdam Institute for Global Health and Development Amsterdam UMC University of Amsterdam Amsterdam the Netherlands; ^8^ Nuffield Department of Medicine University of Oxford Headington United Kingdom

**Keywords:** acute HIV infection, early HIV infection, men who have sex with men, targeted screening, risk score, mobilization, systematic review

## Abstract

**Introduction:**

Screening for acute and early HIV infections (AEHI) among men who have sex with men (MSM) remains uncommon in sub‐Saharan Africa (SSA). Yet, undiagnosed AEHI among MSM and subsequent failure to link to care are important drivers of the HIV epidemic. We conducted a systematic review and meta‐analysis of AEHI yield among MSM mobilized for AEHI testing; and assessed which risk factors and/or symptoms could increase AEHI yield in MSM.

**Methods:**

We systematically searched four databases from their inception through May 2020 for studies reporting strategies of mobilizing MSM for testing and their AEHI yield, or risk and/or symptom scores targeting AEHI screening. AEHI yield was defined as the proportion of AEHI cases among the total number of visits. Study estimates for AEHI yield were pooled using random effects models. Predictive ability of risk and/or symptom scores was expressed as the area under the receiver operator curve (AUC).

**Results:**

Twenty‐two studies were identified and included a variety of mobilization strategies (eight studies) and risk and/or symptom scores (fourteen studies). The overall pooled AEHI yield was 6.3% (95% CI, 2.1 to 12.4; I^2^ = 94.9%; five studies); yield varied between studies using targeted strategies (11.1%; 95% CI, 5.9 to 17.6; I^2^ = 83.8%; three studies) versus universal testing (1.6%; 95% CI, 0.8 to 2.4; two studies). The AUC of risk and/or symptom scores ranged from 0.69 to 0.89 in development study samples, and from 0.51 to 0.88 in validation study samples. AUC was the highest for scores including symptoms, such as diarrhoea, fever and fatigue. Key risk score variables were age, number of sexual partners, condomless receptive anal intercourse, sexual intercourse with a person living with HIV, a sexually transmitted infection, and illicit drug use. No studies were identified that assessed AEHI yield among MSM in SSA and risk and/or symptom scores developed among MSM in SSA lacked validation.

**Conclusions:**

Strategies mobilizing MSM for targeted AEHI testing resulted in substantially higher AEHI yields than universal AEHI testing. Targeted AEHI testing may be optimized using risk and/or symptom scores, especially if scores include symptoms. Studies assessing AEHI yield and validation of risk and/or symptom scores among MSM in SSA are urgently needed.

## 1 INTRODUCTION

In 2018, sub‐Saharan Africa (SSA) faced approximately one million new HIV infections [1]. Although HIV disproportionally affects men who have sex with men (MSM) globally [2, 3], HIV testing and treatment cascade estimates among African MSM are well below target goals set by UNAIDS [4].

HIV incidence estimates among MSM in sub‐Saharan Africa (SSA) are 10 to 15 fold higher than in general populations in Africa: ranging from 5.1/100 person years (PY) (95% confidence interval [CI], 2.6 to 9.8) in Kenya to 12.5/100 PY (95% CI, 8.1 to 19.2) in South Africa and 15.4/100 PY (95% CI, 12.3 to 19.0) in Nigeria [5, 6, 7]. An important driver in the ongoing HIV epidemic among MSM in SSA could be acute and early HIV infections (AEHI), as high viral loads during AEHI lead to a high probability of transmission [8, 9]. Therefore, AEHI is important to diagnose and treat to mitigate onward transmission risk in MSM [10]. Furthermore, immediate treatment after identification of AEHI restores the immune function of people with AEHI [11, 12, 13, 14].

Acute HIV infection (AHI) is typically defined as the first weeks after HIV acquisition, during which HIV antibodies are undetectable [15]. AHI can be diagnosed with HIV‐RNA testing using nucleic acid amplification testing (NAAT) and/or HIV p24‐antigen testing [16, 17]. Early HIV infection (EHI) is usually defined as the first months after HIV acquisition [18, 19]. In this period, HIV antibody tests are often indeterminate. Therefore, diagnosis of EHI requires a combination of HIV antibody, HIV‐RNA, and/or p24 assays [8, 18, 19, 20]. While AEHI testing, here defined as testing with a combination of HIV antibody, HIV‐RNA and p24 assays, was not available in most of SSA until recently, the emergence of point‐of‐care HIV‐RNA testing in SSA enables AEHI testing among a range of populations [21]. In some well‐resourced countries, national guidelines recommend AEHI testing for people who report risk behaviour and symptoms associated with AEHI [22, 23], and facility‐based AEHI testing with HIV‐RNA can successfully identify AEHI among MSM [16, 24, 25, 26, 27, 28, 29]. Unfortunately, global policies do not recommend AEHI testing for MSM [30].

Modelling and phylogenetic transmission studies suggest that 10% to 50% of HIV transmission events occur during AEHI [8, 31, 32, 33, 34, 35]. In order to reduce HIV incidence among MSM, screening strategies should target MSM with the highest risk behaviour, as AEHI yield will be the highest [36]. Ideally, all people at risk of HIV acquisition should be tested for AEHI. However, this may not be feasible in less‐resourced settings due to the high costs of AEHI testing. Focussing on yield would therefore limit the number of people that require AEHI testing, while increasing the number of people diagnosed with AEHI [36]. Behaviour risk scores can identify MSM with high‐risk behaviour [37, 38]. Thus, risk and/or symptom scores may assist in defining which subpopulations should be targeted for AEHI testing [39, 40].

Recently, a systematic review assessed strategies to increase HIV testing among MSM [41]. Authors concluded that social network‐based strategies, community‐based testing, HIV self‐testing and modifications to the traditional facility‐based model can effectively reach urban MSM. However, AEHI testing strategies were not reviewed. The aim of this study was to conduct a systematic review and meta‐analysis of (1) AEHI yield among MSM mobilized for AEHI testing; and (2) assess which risk factors and/or symptoms could increase AEHI yield in MSM.

## 2 METHODS

The Preferred Reporting Items for Systematic Reviews and Meta‐Analysis (PRISMA)‐statement was followed, which provides items for reporting in systematic reviews and meta‐analyses [42].

### 2.1 Search strategy

On 25 May 2020, we searched PubMed, Embase.com, Clarivate Analytics/Web of Science Core Collection and Ebsco/ERIC using search terms, including synonyms and related terms, and keywords such as “men who have sex with men,” “homosexuality,” “acute HIV infection,” “early HIV infection,” and “mobilization” from database inception to the search date mentioned earlier, without geographical or language restrictions. The keywords represented three domains: domains one and two identified studies pertaining to MSM and AEHI respectively. The third domain sought to capture studies that focused on mobilization strategies, which included methods of communication with MSM. The full search strategy is described in Table S1. Experts in the field and secondary reference searching on included studies identified additional studies.

### 2.2 Inclusion criteria and screening

Studies were included when the following inclusion criteria were met: (1) the study described a strategy of mobilizing MSM for AEHI testing; or (2) the study described the development or validation of a risk and/or symptom score which could increase the yield of AEHI in MSM. Studies were excluded if they merely assessed knowledge of AEHI among MSM, assessed AEHI laboratory testing techniques, described AEHI testing among MSM who had already presented for HIV testing, did not include the number of AEHI cases, or described AEHI testing among MSM who had already presented for HIV testing (e.g. laboratory evaluations of pooled samples obtained from MSM who had tested for HIV). Peer‐reviewed articles and conference abstracts were included. For each conference abstract meeting the inclusion criteria, a specific search was set out to identify the subsequent peer‐reviewed article of the study, as such, no conference abstracts were included in the final review. Two independent reviewers (SP and MD) used rayyan.qcri.org to screen titles and abstracts of records identified through the search to remove non‐relevant records. Full‐text records were then assessed for eligibility. Discrepancies were resolved by discussion with a third and fourth reviewer (EJS and GJB). We assessed study quality using the Appraisal tool for Cross‐sectional Studies (AXIS; Table S2) [43].

### 2.3 Data extraction

Data were extracted by two independent reviewers (SP and MD) using a standardized form. If studies reported on both MSM and other populations, we extracted data for MSM only if disaggregated data were available, otherwise we included estimates of the whole sample. We contacted study authors when additional information was needed. A modified framework from Campbell et al. was applied [41]. Studies were categorized according to two principal testing categories: (1) mobilization for AEHI testing, and (2) risk and/or symptom score screening. Mobilization for AEHI testing included three subcategories: media campaigns, partner notification services (PNS) and community‐based testing. The data extracted included the following: AEHI cases identified, the total number of visits during which AEHI was assessed, year of publication, year of conduct, country, study population and study design. For the papers concerning mobilization strategy, we extracted the mobilization strategy, eligibility criteria for AEHI testing, and AHI and EHI definitions. For risk and/or symptoms scores a list of risk factors and/or symptoms included in the score, the recall period, cut‐off value of the score, the area under the receiver operator curve (AUC), sensitivity and specificity of the score.

### 2.4 Mobilization for acute and early HIV infection testing

In literature, different definitions are being used for AEHI based on the interval between infection and evolution of HIV tests as well as dynamics in antibodies over time. We used AEHI definitions as proposed by authors of the included studies. These varying definitions may have biased the cumulative results of this systematic review, however, we were unable to standardize AEHI definitions across the included studies as study authors reported results based on the above‐described definitions. We defined AEHI yield as the proportion of identified AEHI cases among the number of visits during which AEHI was assessed. Targeted AEHI testing was defined as testing among a selected subgroup of MSM based on high‐risk behaviour and/or AEHI symptoms. This was opposed to universal AEHI testing, defined as testing all MSM. Outcomes included type of mobilization strategy, and AHI and AEHI yield.

### 2.5 Data analysis

We pooled independent study estimates for AEHI yield using the Freeman‐Tukey double arcsine transformation in random effects models based on the method of DerSimonian and Laird [44, 45]. Exact binomial procedures were used to calculate 95% CIs [46]. Pooled estimates were back‐transformed on their original scale. Heterogeneity across estimates was assessed using the I^2^ statistic [47]. After observing large heterogeneity across the estimates, we performed sub‐group analyses of studies assessing targeted AEHI and AHI testing and studies assessing universal AEHI and AHI testing. Analyses were performed using the Metaprop package [48] in Stata (version 15.1; StataCorp).

### 2.6 Risk and/or symptom score screening

Outcomes included AUC, sensitivity and specificity for risk and/or symptom scores. We extracted (or calculated, if not provided by authors) sensitivity and specificity at the score cut‐off as proposed by the authors of included studies. We defined internal validation as assessment of predictive ability (AUC, sensitivity and specificity) of a risk and/or symptom score in a different study sample from the same location as the study sample in which the score was developed (i.e. the dataset was randomly split in a development and validation dataset or split based on calendar year). We defined external validation as assessment of predictive ability of a risk and/or symptom score in a study sample from a different location as the study sample in which the score was developed.

## 3 RESULTS

### 3.1 Study selection

**Figure 1:**
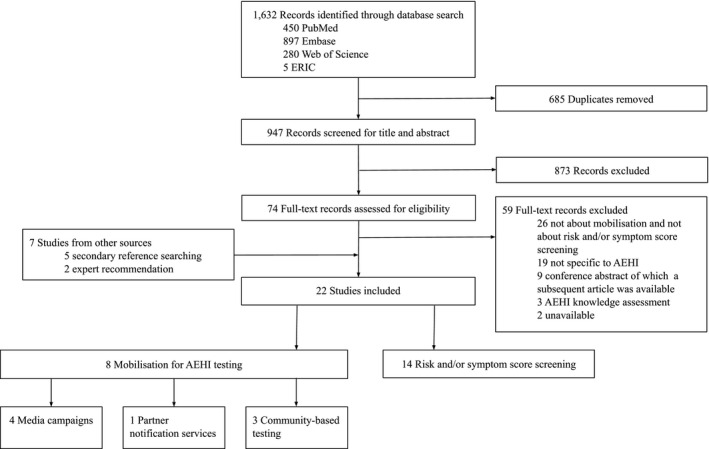
Study selection. AEHI, acute and early HIV infection; ERIC, Education Resources Information Center; MSM, men who have sex with men.

We identified 1632 records through the database search (Figure 1). Following the removal of 685 duplicates, 947 records were screened for title and abstract. Of these, 873 non‐relevant records were excluded and 74 full‐text records were assessed for eligibility, of which 15 records met the eligibility criteria and were included in this study. Seven additional records were identified from other sources: five from secondary reference searching [38, 49, 50, 51, 52] and two from expert recommendation [53, 54]. Taken together, 22 records met the inclusion criteria: eight studies concerned strategies mobilizing MSM for AEHI testing [51, 55, 56, 57, 58, 59, 60, 61] and another 14 studies dealt with risk and/or symptom score screening [17, 37, 38, 39, 40, 49, 50, 52, 53, 54, 62, 63, 64, 65]. Critical appraisal showed that none of the included studies justified their sample size and most studies did not address, categorize or describe information about non‐responders (Table S3).

### 3.2 Characteristics of mobilization studies

Of the eight studies that assessed strategies mobilizing MSM for AEHI testing, seven studies originated from well‐resourced settings [51, 55, 56, 57, 58, 59, 61]. One study originated from a less‐resourced setting and was conducted in Thailand [60] (Table 1). All eight studies were cross‐sectional studies and were conducted between 1996 and 2017 [51, 55, 56, 57, 58, 59, 60, 61]. Seven studies exclusively targeted MSM [55, 56, 57, 58, 59, 60, 61]. One study included sexual or injection drug equipment partners of people living with HIV (PLWH) [51]. Although this study did not specify the number of MSM included, they predominantly targeted MSM during recruitment.

**Table 1:** Studies assessing strategies to mobilize men who have sex with men for testing for acute and early HIV infection

First author	Media campaigns				Partner notification services	Community‐based testing		
	Silvera	Stekler	Gilbert	Dijkstra	Green	Daskalakis	Liang	Pankam
Site	New York City [51]	Seattle [56]	Vancouver [57]	Amsterdam [61]	San Diego [58]	New York City [55]	Hong Kong [59]	Bangkok [60]
Country	USA	USA	Canada	The Netherlands	USA	USA	Hong Kong	Thailand
Years study conducted	2004 to 2008	2004 to 2009	2006 to 2012	2008 to 2017	1996 to 2014	2007	2010 to 2011	2011 to 2012
Year of publication	2010	2013	2013	2020	2017	2009	2015	2018
Study design	Cross‐sectional	Cross‐sectional	Cross‐sectional	Cross‐sectional	Cross‐sectional	Cross‐sectional	Cross‐sectional	Cross‐sectional
Target population	Heterosexual men and women, MSM	MSM	MSM	MSM	MSM	MSM	MSM	MSM
Mobilization strategy	Media campaign	Media campaign	Media campaign	Media campaign	PNS	MSM venue‐based testing^a^	MSM venue‐based testing^b^	MSM venue‐based testing^c^
Eligibility criteria for AEHI testing	Sex or sharing injection drug equipment with a PLWH^d^, ^e^	MSM presenting for HIV testing	Men, TGP presenting for HIV testing^f^	ARS and CAI^e^	MSM^g^ partners of people with AEHI	MSM venue visitors	MSM venue visitors	MSM venue visitors and reporting sex with men^h^
Targeted AEHI testing	Yes	No	No	Yes	Yes	No	No	No
AHI definition								
RNA	+^i^	+^j^	+^k^	+^l^	+^m^	+^n^	+^o^	+^p^
Ag/Ab	NP	NP	− or + ^q^	− or ±^r^	NP	NP	NP	− or + r
Ab	− or + ^s^	−^t^	−^t^	−^u^	− or ±^u^	−^u^	−^u^	−^t^, ^u^
WB	− or ± or + ^v^	−	−	−	NP	−	NP	NP
EHI definition	Infection <129 days^w^	NS	NS	WB− or WB±	Infection <170 days^w^	Seroconversion <170 days^w^	Seroconversion <6 months^x^	NS

Ab, second or third generation rapid antibody test; AEHI, acute and early HIV infection; Ag/Ab, fourth generation antigen/antibody test; AHI, acute HIV infection; ARS, acute retroviral syndrome; CAI, condomless anal intercourse; EHI, early HIV infection; MSM, men who have sex with men; NP, not performed; NS, not specified; PLWH, person living with HIV; PNS, partner notification services; RNA, ribonucleic acid; TGP, transgender people; USA, United States of America; WB, western blot.

^a^ MSM bathhouses

^b^ MSM bars, saunas, clubs and a local non‐governmental organization

^c^ MSM saunas and spa venues

^d^ or of unknown HIV status

^e^ in the previous three months

^f^ or if sex was missing

^g^ the original study did not report solely on MSM, disaggregated data on MSM partners (as reported here) were provided by the authors

^h^ at least once in their lifetime

^i^ HIV plasma viral load

^j^ pooled HIV nucleic acid

^k^ <2009: HIV nucleic acid; ≥2009: Pooled HIV nucleic acid

^l^ point‐of‐care HIV‐RNA

^m^ <2007: Quantitative HIV‐RNA; ≥2007: HIV nucleic acid

^n^ pooled HIV viral load

^o^ point‐of‐care real‐time dried blood spot‐based quantitative polymerase chain reaction

^p^ HIV nucleic acid, HIV viral load

^q^ <2009: p24 antigen, discontinued from 2009

^r^ fourth generation antigen/antibody

^s^ Ezyme‐linked immunosorbent assay

^t^ Enzyme immunoassay

^u^ rapid antibody

^v^ or a documented negative antibody test in the previous 30 days

^w^ estimated by recency assays or a serologic testing algorithm for recent seroconversion[18, 19]

^x^ positive rapid antibody test with self‐reported negative antibody test in the previous six months.

### 3.3 Strategies for mobilization for acute and early HIV infection testing

The eight studies that assessed strategies mobilizing MSM for AEHI testing included four studies assessing the impact of media campaigns [51, 56, 57, 61], one describing PNS for people with AEHI [58], and three describing community‐based testing for AEHI [55, 59, 60]. Three studies reported on targeted AEHI testing [51, 58, 61] and five studies on universal AEHI testing [55, 56, 57, 59, 60].

Media campaigns aimed to target MSM to increase knowledge and awareness of AEHI, the increased transmission risk, AEHI symptoms, AEHI tests and early treatment. Furthermore, they aimed to increase motivation to test for AEHI and included referral for facility‐based AEHI testing. The campaigns were developed and promoted in conjunction with MSM community‐based organizations [51, 56, 57, 61]. Resources included print advertisements, condom packs, billboards, posters, web‐based advertisements (e.g. on dating websites and applications) and campaign websites. These were promoted at MSM community‐based events and MSM venues such as bars and bathhouses, MSM‐targeted magazines and HIV testing facilities.

One study offered PNS to people with AEHI (index clients) [58]. The target population included MSM sexual or injection drug equipment partners of index clients with AEHI. Referral was done by index clients, with or without assistance of a healthcare provider, or by a healthcare provider without disclosing the identity of the index client.

Three studies assessed community‐based AEHI testing at MSM venues [55, 59, 60]. The target population consisted of MSM visiting the venues. Venues included bathhouses, saunas, spas, bars, clubs and local non‐governmental organizations. Collection of samples, conduction of rapid antibody tests and delivery of rapid antibody test results took place on‐site at the venues. AEHI testing was laboratory based.

### 3.4 Definitions of acute and early HIV infection

AHI was defined as a positive HIV‐RNA test and a negative antibody test in six included studies [55, 56, 57, 59, 60, 61], as a positive HIV‐RNA test and an indeterminate antibody test in one study [58], or as a positive HIV‐RNA test and a positive antibody test and a documented negative antibody test in the previous 30 days in one study [51]. Five included studies defined and reported on EHI, varying from a negative or indeterminate Western blot test to a documented or self‐reported negative antibody test in the previous six months [51, 55, 58, 59]. HIV tests included (pooled) HIV plasma viral load, point‐of‐care HIV‐RNA tests, fourth generation antigen/antibody tests, rapid antibody tests and Western blot.

### 3.5 Acute and early HIV infection yield

**Figure 2:**
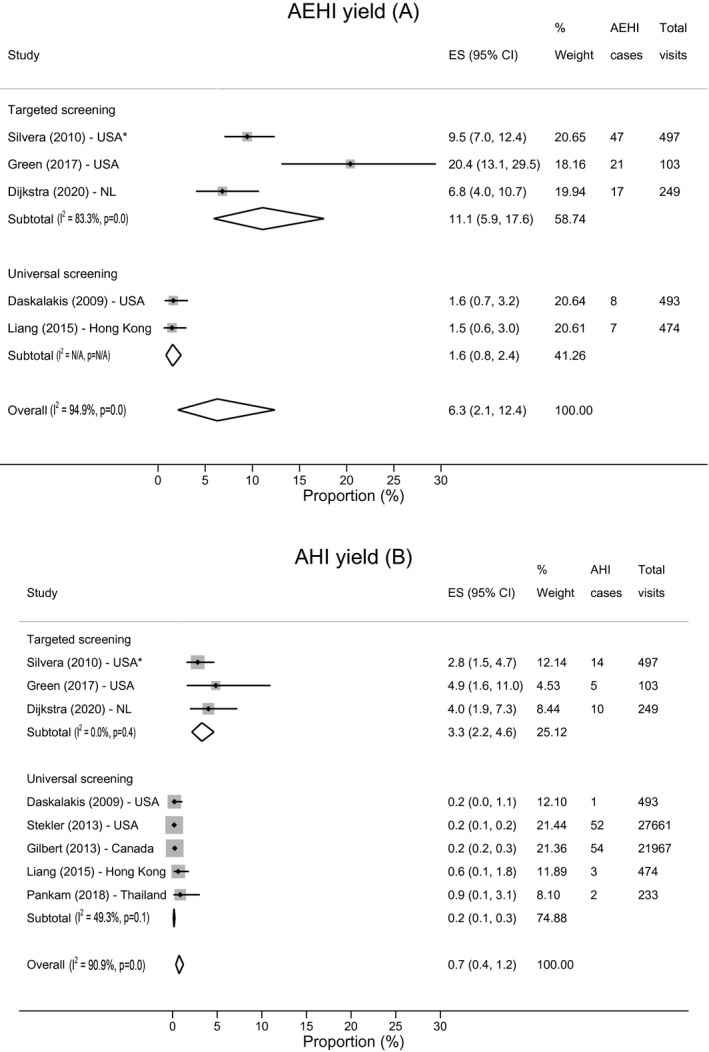
Forest plots of acute HIV infection yield and acute and early HIV infection yield among men who have sex with men. Study estimates and their 95% CIs, and pooled estimates and their 95% CIs for AEHI yield, overall and stratified by testing strategy: targeted testing and universal testing. **(A)** Displays AEHI yield, **(B)** displays AHI yield. Yield was defined as the proportion of AEHI cases among the number of visits during which AEHI was assessed. The size of the grey boxes represents a study’s weight in the meta‐analysis. *The study population was men who have sex with men in all studies, with the exception of Silvera et al. In this study, heterosexual men, women and men who have sex with men were included, however, they predominantly targeted MSM during recruitment. AHI, acute HIV infection; AEHI, acute and early HIV infection; CI, confidence interval; ES, effect size; N/A, not accessible; NL, the Netherlands; USA, United States of America.

The above‐described mobilization strategies resulted in a pooled AEHI yield of 6.3% (95% CI, 2.1 to 12.4; I^2^ = 94.9%; five studies [51, 55, 58, 59, 61]); this was 11.1% (95% CI, 5.9 to 17.6; I^2^ = 83.8%) among the three studies assessing targeted testing [51, 58, 61], and 1.6% (95% CI, 0.8 to 2.4) among the two studies assessing universal testing [55, 59] (Figure 2).

### 3.6 Acute HIV infection yield

The overall pooled AHI yield was 0.7% (95% CI, 0.4 to 1.2; I^2^ = 90.9%; eight studies) [51, 55, 56, 57, 58, 59, 60, 61]. Among the three studies assessing targeted testing, the pooled AHI yield was 3.3% (95% CI, 2.2 to 4.6; I^2^ = 0%) [51, 58, 61], and among the five studies assessing universal testing this was 0.2% (95% CI, 0.1 to 0.3; I^2^ = 49.3%) [55, 56, 57, 59, 60]. The highest AHI yield was recorded in a study among MSM partners of people with AEHI: 4.9% (95% CI, 1.6 to 11.0) [58]. Three studies assessed whether implementation of the media campaign led to increased AHI yield compared with pre‐implementation: AHI yield increased in Vancouver and Amsterdam post‐implementation, but not in Seattle [56, 57, 61]. This assessment was quantified by two studies, therefore, we included post‐implementation estimates in the pooled analysis [57, 61].

### 3.7 Characteristics of risk and/or symptom score studies

Of the 14 studies that assessed risk and/or symptom score screening, 11 studies originated from well‐resourced settings [37, 39, 40, 49, 50, 52, 54, 62, 63, 64, 65] (Table 2). The three studies from less‐resourced settings originated from Kenya [17, 38, 53]. There were four cross‐sectional studies [39, 40, 62, 64], seven prospective cohort studies [17, 37, 38, 53, 54, 63, 65], one retrospective cohort study [52], one study analysed both cross‐sectional data and data from a randomized controlled trial (RCT) [49], and one study analysed data solely originating from RCTs [50]. These studies used datasets collected between 1984 and 2018. Twelve studies exclusively included MSM [17, 37, 38, 39, 49, 50, 52, 53, 54, 63, 64, 65] and two studies focused on people who had presented for HIV testing (e.g. clients of sexually transmitted infection [STI] clinics) [40, 62], of which MSM were the vast majority (>70%) of participants.

**Table 2:** Overview of published risk and/or symptom scores to assist screening for acute and early HIV infection among men who have sex with men

First author	Years study conducted	Year of publication	Site	Country	Study design	Study population	Score name^a^	Development (D) and/or validation (V)
Menza [49]	1999 to 2008	2009	Boston, Chicago, Denver, New York, San Francisco, Seattle	USA	Cross‐sectional/ RCT	MSM	Menza	D and V
Facente [62]	2004 to 2007	2011	San Francisco	USA	Cross‐sectional	STI clinic clients	Facente	D and V
Smith [50]	1998 to 2001	2012	57 cities	USA	RCT	MSM^b^	HIRI‐MSM	D and V
Wahome [38]	2005 to 2012	2013	Kilifi	Kenya	Prospective cohort	MSM	CDRSS	D
							UMRSS^c^	V
Hoenigl [39]	2008 to 2014	2015	San Diego	USA	Cross‐sectional	MSM	SDET	D and V
							HIRI‐MSM	V
							Menza	V
Sanders [53]	1993 to 2012	2015	Kilifi	Kenya	Prospective cohort	MSM	Sanders	D
Beymer [52]	2009 to 2014	2017	Los Angeles	USA	Retrospective cohort	MSM	Beymer	D
Jones [63]	2010 to 2014	2017	Atlanta	USA	Prospective cohort	MSM	SDET	V
							HIRI‐MSM	V
							Menza	V
Dijkstra [65]	1984 to 2009	2017	Amsterdam, Baltimore, Chicago, Pittsburg, Los Angeles	The Netherlands, USA	Prospective cohort	MSM	Amsterdam score	D and V
Lancki [54]	2013 to 2016	2018	Chicago	USA	Prospective cohort	MSM	CDC	V
							HIRI‐MSM	V
							Gilead	V
Wahome [17]	2005 to 2016	2018	Kilifi	Kenya	Prospective cohort	MSM	Wahome	D
Lin [40]	2007 to 2017	2018	San Diego	USA	Cross‐sectional	STI clinic clients^d^	SDSS	D and V
Lin [64]	2007 to 2017	2018	San Diego	USA	Cross‐sectional	MSM	Amsterdam score	V
Dijkstra [37]	2003 to 2018	2019	Amsterdam	The Netherlands	Prospective cohort	MSM	SDET	V

CDC, Centers For Disease Control and Prevention; CDRSS, Cohort Derived Risk Screening Score; D, development; HIRI‐MSM, HIV Incidence Risk Index for MSM; MSM, men who have sex with men; RCT, randomized controlled trial; SDET, San Diego Early Test; SDSS, San Diego Symptom Score; STI, sexually transmitted infection; UMRSS, University of North Carolina Malawi Risk Screening Score; USA, United States of America; V, validation.

^a^ 14 studies assessed predictive ability of 13 independent risk and/or symptom scores, five scores were assessed multiple times

^b^ 75.0% (9472/12622) of participants were MSM

^c^ The development study of the UMRSS was not included in this review, as it did not include MSM

^d^ 73.8% (737/998) of participants were MSM.

### 3.8 Risk and/or symptom score screening

The 14 studies assessed predictive ability of 13 independent risk and/or symptom scores to target AEHI testing among MSM [17, 37, 38, 39, 40, 49, 50, 52, 53, 54, 62, 63, 64, 65]. In total, the 14 studies included 26 score outcomes (including AUC, sensitivity and specificity from nine development and 17 validation outcomes), as most scores were assessed multiple times (Table 3). Four scores were not validated [17, 38, 52, 53].

**Table 3:** Point values of risk factors and symptoms included in published risk and/or symptom scores to assist screening for acute and early HIV infection among men who have sex with men

Score name	Menza [49]	Facente [62]	HIRI‐MSM [50]	CDRSS [38]	UMRSS [38]	SDET [39]	Sanders [53]	Beymer [52]	Amsterdam score [65]	CDC [54]	Gilead [54]	Wahome [17]	SDSS [40]
Recall period	6 to 12 months	2 years^a^	6 months	4 to 12 weeks	4 to 12 weeks	12 months	4 to 12 weeks	1 to 12 months	6 months	6 months	NS	1 to 12 weeks	2 weeks
Cutoff	≥1	≥2	≥10	≥2	≥2	≥5	≥2	≥5^b^	≥1.5	≥1^c^	≥1	≥1	≥11
Risk or symptom score	Risk	Risk	Risk	Risk/symptom	Risk/symptom	Risk	Risk/symptom	Risk	Risk/symptom	Risk	Risk	Risk	Symptom
Point values													
Risk factors													
Age			2 to 8^d^	1^e^			1^e^	0.27 to 0.48^f^				1^g^	
Ethnicity								0.27 to 0.68^h^					
MSM		1											
Sex with only men												1	
IDU		1											
Incarceration											1		
No. of partners	3^i^		4 to 7^j^		1^k^	2^i^		0.01^l^	0.9^m^				
Partner characteristics								0.005 to 0.45^n^		1^o^			
IPV								0.31					
RAI								0.35^p^				1	
CI											1	1	
CRAI		1	10			3^q^		0.61	1.1	1^r^			
HIV + partner		1	4 to 8^s^							1			
CAI with HIV + partner	1		6^t^			3^u^							
Group sex												1	
Transactional sex											1		
Self‐reported STI	4	1^v^		1		2		0.19 to 0.75^w^	1.6^x^	1	1		
Methamphetamine use	11^y^		5					0.49			1^z^		
Inhaled nitrites			3					0.45					
Ecstasy use								0.21					
Discordant HIV rapid antibody tests				4	4								
Symptoms													
Body pains/ myalgia							1						8
Diarrhoea				1	2		1						
Fever				1	1		1		1.6				11
Fatigue				1	2		1						
Genital ulcers							3						
Lymphadenopathy									1.5				
Oral thrush									1.7				
Sore throat							1						
Weight loss									0.9				4^aa^
Number of validations													
Internal^bb^	0	1	0	0	0	1	0	0	0	0	0	0	1
External^cc^	3	0	4	0	1	2	0	0	2	1	1	0	0

CAI, condomless anal intercourse; CDC, Center for Disease Control and Prevention; CDRSS, Cohort Derived Risk Screening Score; CI, condomless intercourse; CRAI, condomless receptive anal intercourse; HIRI‐MSM, HIV Incidence Risk Index for MSM; HIV+, HIV‐infected; IDU, injection drug use; IPV, intimate partner violence; MSM, men who have sex with men; NS, not specified; RAI, receptive anal intercourse; SDET, San Diego Early Test; SDSS, San Diego Symptom Score; STI, sexually transmitted infection; UMRSS, UNC Malawi Risk Screening Score.

^a^ Or since last HIV test

^b^ for all risk and/or symptom scores, the point values of the variables in the score were summed to obtain an individual’s score, except for Beymer’s score: the point values were added and then exponentiated

^c^ an individual’s score was only assessed if he reported any male sex partners in previous six months, was not in a monogamous partnership with a recently tested or HIV‐uninfected man

^d^ 18 to 28 years = 2 points, 29 to 40 years = 5 points, 41 to 48 years = 2 points

^e^ 18 to 29 years

^f^ <25 years = 0.48 points, 25 to 29 years = 0.36 points, 30 to 39 years = 0.27 points

^g^ 18 to 24 years

^h^ black = 0.68 points, Hispanic = 0.52 points, other = 0.27 points

^i^ >9 partners

^j^ 6 to 10 partners = 4 points, >10 partners = 7 points

^k^ >1 partners

^l^ ≤3 or> 3 partners

^m^ >5 partners

^n^ >age of last sex partner five years older; within five years of age; or >5 years younger = 0.005 points, same ethnicity as last partner = 0.45 points

^o^ partners of unknown HIV status with any of the following factors: inconsistent or no condom use, STI, transactional sex, use of illicit drugs or alcohol dependence, incarceration

^p^ RAI with a condom

^q^ CRAI and >4 partners

^r^ any condomless anal sex (insertive or receptive)

^s^ 1 HIV‐infected partner = 4 points; >1 HIV‐infected partners = 8 points

^t^ condomless insertive anal intercourse with >5 HIV‐infected partners

^u^ condomless receptive anal intercourse with an HIV‐infected partner

^v^ a simplified model without STI had similar performance but was not included in this review

^w^ diagnosed with an STI > 1 year ago = 0.19 points, <1 year ago = 0.75 points

^x^ self‐reported gonorrhoea

^y^ or use of inhaled nitrites

^z^ use of illicit drugs or alcohol dependence (excluding marijuana)

^aa^ ≥2.5 kg

^bb^ assessment of predictive ability of the score in a different study sample from the same location as the study sample in which the risk and/or symptom score was developed

^cc^ assessment of predictive ability of the risk and/or symptom score in a study sample from a location different to the study sample in which the score was developed.

### 3.9 Variables included in risk and/or symptom scores

The recall period for risk factors and symptoms included in the scores varied from two weeks to two years. The 13 scores comprised eight scores only including demographic or behavioural risk factors for HIV acquisition [17, 39, 49, 50, 52, 54, 62], four scores including risk factors and AEHI symptoms [38, 53, 65] and one score including only AEHI symptoms [40] (Table 3). Most frequently included risk factors were age, number of sexual partners, condomless receptive anal intercourse (CRAI), sexual intercourse with a PLWH, self‐reported diagnosis of an STI and illicit drug use. Most frequently included symptoms were self‐reported diarrhoea, fever and fatigue [17, 38, 40, 53, 65]. Three scores were incorporated in MSM‐targeted websites, to allow for self‐assessment of HIV risk (www.hebikhiv.nl/en; www.IsPrEPforMe.org; http://sdet.ucsd.edu [39, 52, 61]).

### 3.10 Predictive ability of the risk and/or symptom scores

**Figure 3:**
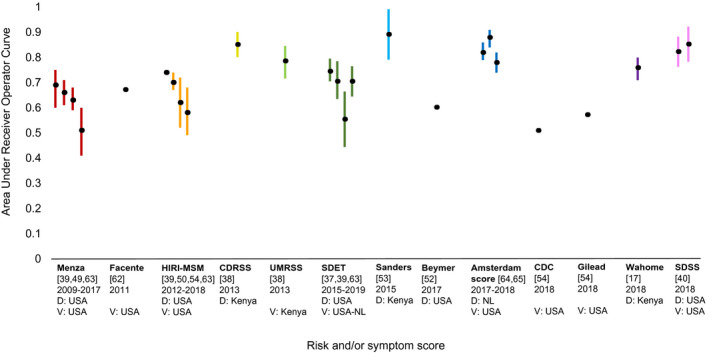
Area under receiver operator curves of published risk and/or symptom scores to assist screening for acute and early HIV infection among men who have sex with men. The black dots represent point estimates, the coloured lines 95% confidence intervals. If no coloured lines are displayed, the study did not report 95% confidence intervals. For each risk and/or symptom score, the first point estimate represents the area under receiver operator curve of the development study sample, the latter point estimate(s) represents the area under receiver operator curve of the validation study sample(s). The development outcomes of scores Facente, UMRSS, CDC and Gilead have not been included in this review, therefore, only validation outcomes are represented. CDC, Center for Disease Control and Prevention; CDRSS, Cohort Derived Risk Screening Score; D, Development study sample; HIRI‐MSM, HIV Incidence Risk Index for MSM; MSM, men who have sex with men; NL, the Netherlands; NS, not specified; SDET, San Diego Early Test; SDSS, San Diego Symptom Score; UMRSS, University of North Carolina Malawi Risk Screening Score; USA, United States of America; V, Validation study sample.

The AUC ranged from 0.69 to 0.89 in development study samples, and from 0.51 to 0.88 in validation study samples (Table 4 and Figure 3). Sensitivity at the cut‐off proposed by the authors ranged from 74% to 98% in development study samples, and from 25% to 94% in validation samples. Specificity was between 17% and 90% in development study samples, and between 15% and 96% in validation study samples.

**Table 4:** Predictive ability of published risk and/or symptom scores to assist screening for acute and early HIV infection among men who have sex with men

First author	Score name^a^	Total visits (n)		AEHI cases (n)		AUC (95% CI)		Sensitivity (%)^b^		Specificity (%)^b^	
		D	V	D	V	D	V	D	V	D	V
Menza [49]	Menza	NS	NS	101	104	0.69 (0.60 to 0.74)	0.66 (0.61 to 0.71)	83%	86%	30%	29%
Facente [62]	Facente	12,350	12,249^c^	137	36		0.67 (NS)		83%		50%
Smith [50]	HIRI‐MSM	24,391	15,582	320	171	0.74 (NS)	NS	84%	81%	45%	38%
Wahome [38]	CDRSS	6531		73		0.85 (0.80 to 0.90)		81%		76%	
	UMRSS		6531		73		0.79 (0.72 to 0.85)		75%		76%
Hoenigl [39]	SDET	5568	2758	137	63	0.74 (0.70 to 0.79)	0.70 (0.63 to 0.78)	NS	60%	NS	77%
	HIRI‐MSM		8326		200		0.70 (0.67 to 0.74)		69%		60%
	Menza		8326		200		0.63 (0.59 to 0.68)		67%		54%
Sanders [53]	Sanders	7054		20		0.89 (0.79 to 0.99)		74%		90%	
Beymer [52]	Beymer	NS		370		0.6 (NS)		75%		50%	
Jones [63]	SDET		3372		32		0.55 (0.44 to 0.66)		25%		84%
	HIRI‐MSM		372		32		0.62 (0.52 to 0.72)		63%		57%
	Menza		3372		32		0.51 (0.41 to 0.60)		63%		41%
Dijkstra [65]	Amsterdam score	17,446	63,618	175	491	0.82 (0.79 to 0.86)	0.78 (0.74 to 0.82)	76%	56%	76%	89%
Lancki [54]	CDC		866		33		0.51 (NS)		52%		52%
	HIRI‐MSM		866		33		0.580.49 to 0.68		85%		30%
	Gilead		866		33		0.57 (NS)		94%		15%
Wahome [17]	Wahome	9143		97		0.76 (0.71 to 0.80)		98%		17%	
Lin [40]	SDSS	673	325	70	43	0.82 (0.76 to 0.88)	0.85 (0.78 to 0.92)	NS	72%	NS	96%
Lin [64]	Amsterdam score		757		110		0.88 (0.84 to 0.91)		78%		81%
Dijkstra	SDET		14,695				0.70 (0.64 to 0.76)		54%		78%

AEHI, acute and early HIV infection; AUC, area under receiver operator curve; CDC, Center for Disease Control and Prevention; CDRSS, Cohort Derived Risk Screening Score; CI, confidence interval; D, Development study sample; HIRI‐MSM, HIV Incidence Risk Index for MSM; MSM, men who have sex with men; NS, not specified; SDET, San Diego Early Test; SDSS, San Diego Symptom Score; UMRSS, University of North Carolina Malawi Risk Screening Score; V, Validation study sample.

^a^ 13 studies assessed predictive ability of 13 independent risk and/or symptom scores, five scores were assessed multiple times

^b^ at the cutoff specified by the authors

^c^ the HIV negative visits were used in both the development and validation dataset.

Internal and external validation resulted in lower predictive ability for most scores. For example the San Diego Early Test (SDET) score yielded an AUC of 0.74 (95% CI, 0.70 to 0.79) in the development study sample, and between 0.55 (95% CI, 0.44 to 0.66) to 0.70 (95% CI, 0.63 to 0.78) in external validation samples [37, 39, 63]. A study in Atlanta validated three scores (SDET, HIRI‐MSM and the Menza score) in a cohort with a high proportion of HIV seroconversions among Black MSM, whereas the scores had been developed and previously validated in study samples consisting of predominantly white MSM [63]. The three scores performed poorly in this validation study sample among Black MSM and had markedly lower AUC values than in other validation study samples. This was also the case for a validation study in Chicago among young Black MSM [54]. Two scores showed high predictive ability in both the development and validation study samples: the Amsterdam score yielded AUC values of 0.78 (95% CI, 0.74 to 0.82) and 0.88 (95% CI, 0.84 to 0.91) in external validation study samples [64, 65], the San Diego Symptom Score (SDSS) yielded an AUC of 0.85 (95% CI, 0.78 to 0.92) in internal validation [40]. Both scores included symptoms. Other scores, all from Kenya, with high AUC values in development study samples (0.76 to 0.89) have not been validated [17, 38, 53].

## 4 DISCUSSION

In this systematic review and meta‐analysis, we showed substantial AHI and AEHI yields when MSM were mobilized for AEHI testing in studies predominantly conducted in well‐resourced settings. With the severe ongoing HIV epidemic among MSM in SSA [5, 6, 7], infrequent HIV testing and poor linkage to care and viral suppression outcomes [4], there is an urgent need to better identify AEHI in MSM. As such, targeted AEHI testing will likely result in high AEHI yields among MSM in SSA. Unfortunately, the World Health Organization (WHO) has no targeted AEHI testing recommendation for key populations, including MSM who have among the highest incidences [5, 6, 7]. Thus, AEHI testing should be offered to MSM, be supported by specific policy recommendations for MSM, and AEHI testing guidelines tailored to SSA need to be developed and endorsed by WHO.

Strategies mobilizing MSM for targeted AEHI testing resulted in higher AEHI yields than strategies mobilizing MSM for universal AEHI testing. Targeted AEHI testing may be optimized by screening with risk and/or symptom scores. The pooled AEHI yield was the highest when testing was targeted to MSM partners of people with AEHI, to partners of PLWH, or to MSM with AEHI symptoms who reported CRAI (11.1%). Although our review identified one study with a high AEHI yield resulting from PNS [58], two other studies did not assess and report on AEHI yield resulting from PNS for index clients with AEHI, and were therefore not included in this review [66, 67]. When focussing only on AHI, the pooled AHI yield among studies assessing targeted testing was 3.3%.

Collaboration with MSM community‐based organizations was key in successfully mobilizing MSM for AEHI testing, either through the design and promotion of AEHI media campaigns, or through the delivery of community‐based testing [51, 55, 56, 57, 59, 60, 61]. In the studies included in this review, on‐site AEHI diagnosis was not possible in community‐based testing settings, but required laboratory‐based tests and skilled laboratory personnel. The emergence of point‐of‐care HIV‐RNA tests may enable on‐site community‐based AEHI testing in SSA [21]. However, no study approached AEHI testing in a comprehensive, culturally sensitive and integrated fashion in SSA. As such, these strategies need to be urgently developed in close collaboration with local community‐based organizations, including the need to include learning about point‐of‐care HIV‐RNA testing when locally available. While WHO recommends regular HIV testing for MSM, we suggest that MSM with unknown or HIV‐negative status who experience AEHI symptoms or meet risk criteria be evaluated for AEHI, especially when PrEP initiation is considered [68].

Opportunities to diagnose AEHI are often missed, due to the non‐specificity of symptoms and the costly diagnostic assays required for AEHI diagnosis [69, 70, 71, 72]. The studies included in this review used several testing strategies to identify AEHI, including point‐of‐care HIV‐RNA testing and (pooled) HIV viral load testing. A study in San Diego showed that AEHI testing with HIV‐RNA testing was cost‐effective in populations of MSM with an HIV prevalence above 0.4% [73]. Since HIV prevalence in MSM in SSA is estimated to be well above this threshold [2], AEHI testing among SSA MSM may also be cost‐effective, although evidence hereof is lacking. Furthermore, targeting resources to specific subpopulations of MSM (e.g. those reporting high‐risk behaviour and/or symptoms) can substantially reduce costs compared with universal AEHI testing [36].

We identified 13 risk and/or symptom scores that may increase AEHI yield in MSM. Key risk factors included in these scores were age, number of sexual partners, CRAI, sexual intercourse with a PLWH, self‐reported diagnosis of an STI and illicit drug use. Key symptoms were self‐reported diarrhoea, fever and fatigue. As knowledge of symptoms of AEHI among MSM is low [74, 75], these risk factors and symptoms may be used to educate MSM and help them self‐recognize AEHI. Several risk and/or symptom scores have been included in MSM‐targeted websites, facilitating self‐assessment of HIV acquisition risk [39, 52, 61], although outcomes of these self‐assessment tools need to be evaluated.

Predictive ability of the 13 risk and/or symptom scores varied greatly and was highest for scores that included symptoms [40, 53, 64, 65]. Validation showed lower discriminate ability of most risk and/or symptom scores in the validation study sample than in the development study sample [52, 54, 63]. This was specifically the case for validation of risk and/or symptom scores among Black MSM in the USA, as the risk and/or symptom scores poorly predicted HIV acquisition [54, 63]. This underlines the importance of external validation of risk and/or symptom scores [76]. Importantly, none of the MSM risk and/or symptom scores developed in SSA were validated [17, 38, 53]. Furthermore, no risk and/or symptom scores developed in well‐resourced settings have been validated in less‐resourced settings.

Scores including symptoms may be particularly useful in SSA, where stigma and discrimination towards MSM behaviour is high, and social desirability bias may prevent MSM from disclosing high‐risk behaviour to healthcare providers [77, 78, 79]. However, symptoms may vary by HIV‐1 subtype [80], limiting the generalizability of symptom‐based scores across SSA.

Risk‐based scores may assist targeted AEHI screening, but may also be of use in identifying and prioritizing candidates for pre‐exposure prophylaxis (PrEP) [81]. Recent studies using machine learning of routine health care data from electronic patient records to identify potential PrEP candidates among the general population showed high predictive ability of generated risk‐based scores, but included more than 20 variables [82, 83, 84], which may limit practical use. Simpler risk and/or symptom scores, consisting of a smaller number of variables, which requires simple summation to calculate an individual’s score, could be implemented in resource‐limited settings. However, risk and/or symptom scores are imperfect, and using a risk and/or symptom score to define who will be tested for AEHI will inevitably exclude people with AEHI [85, 86]. Thus far, no AEHI yield has been reported resulting from screening MSM with published AEHI risk and/or symptom scores.

This study has some limitations. First, the database search strategy did not identify seven out of 22 included studies. Some of the included studies not identified by the search strategy focused on PrEP screening scores rather than AEHI‐screening scores. Because these scores may also assist AEHI screening, we included these studies in this review. Second, heterogeneity across study estimates was large. This was partly explained by different testing strategies; heterogeneity was smaller when we stratified for testing strategy. Another possible explanation is the variable definitions for AEHI as proposed by study authors. This has possibly overestimated the AHI yield in studies that included indeterminate or positive antibody tests in their AHI definition [51, 58]. Additionally, the variable study designs may have increased heterogeneity. For risk and/or symptom scores, the high variability in recall periods (two weeks to two years) will have likely resulted in variable outcomes. Likewise, the risk and/or symptoms recorded varied considerably between studies depending on the local context and how their data collection was set up, thus impacting the comparability of different scores. Furthermore, studies originated from various locations with different HIV epidemics, which has likely increased heterogeneity. Third, we did not standardize the cutoff at which sensitivity and specificity were assessed for the risk and/or symptom scores, and as a result, these values varied across studies. This has limited the comparison of sensitivities and specificities for the risk and/or symptom scores.

## 5 CONCLUSIONS

In conclusion, strategies mobilizing MSM for targeted AEHI testing resulted in higher AEHI yields than universal AEHI testing. Targeted AEHI testing may be optimized using risk and/or symptom scores, in particular scores including symptoms. However, yield of AEHI testing has not been assessed among MSM in SSA and validation of risk and/or symptom scores among MSM in SSA is urgently needed. With the emergence of point‐of‐care HIV‐RNA testing platforms in SSA, MSM with unknown or HIV‐negative status who have AEHI symptoms or meet AEHI risk behaviour criteria should be evaluated for AEHI. These programmes should be developed in a culturally sensitive fashion, for example through collaborating with local community‐based organizations to promote learning about AEHI symptoms, and or risk behaviour, particularly in SSA. Further studies should focus on AEHI yield and cost‐effectiveness resulting from risk and/or symptom score screening, and the development and validation of culturally sensitive approaches to target MSM for AEHI screening in SSA.

## COMPETING INTERESTS

GJB has received grants through her institution from Bristol‐Meyer Squibbs and Mac Aids Fund; honoraria to her Institution for scientific advisory board participations for Gilead Sciences and speaker fees from Gilead Sciences and Takeda. The remaining authors declared no competing interests.

## AUTHORS’ CONTRIBUTIONS

SP, MD, EW, JW, EG, EME and EJS designed the study. JK designed the search strategy. SP and MD independently assessed records for eligibility, and conducted data extraction, supported by EW. GJB and EJS had oversight in study selection and data extraction. MD conducted the statistical analysis and drafted the manuscript. MFSVL had oversight in the statistical analysis. All authors critically reviewed and revised the manuscript and approved the final version for publication.

## Supporting information


**Table S1.** Database search strategies
**Table S2.** The appraisal tool for cross‐sectional studies
**Table S3.** Critical appraisal of included studies using the Appraisal Tool For Cross‐Sectional Studies
