# Supplementary material for: Acute and early HIV infection screening among men who have sex with men, a systematic review and meta‐analysis
Source: J Int AIDS Soc. 2020 Oct 1;23(Suppl 6):e25590. doi: 10.1002/jia2.25590 (PMC7527764; doi:10.1002/jia2.25590)
Supplement: Supplementary file 1 — Table S1. Database search strategies Table S2. The appraisal tool for cross‐sectional studies Table S3. Critical appraisal of included studies using the Appraisal Tool For Cross‐Sectional Studies [file JIA2-23-e25590-s001.docx]

Table S1. Database search strategies

| **Database and search date** | **Number of items found** |
| --- | --- |
| PubMed 25 May 2020 | 450 |
| Embase.com 25 May 2020 | 897 |
| Web of Science 25 May 2020 | 280 |
| ERIC 25 May 2020 | 5 |
| **Total** | 1632 |

# PubMed 25 May 2020

| **Search** | **Query** | **Items** |
| --- | --- | --- |
| **#5** | #4 NOT *[401 PMIDs]* | **49** |
| **#4** | #1 AND #2 AND #3 | **450** |
| **#3** | "Homosexuality"[Mesh:NoExp] OR "Homosexuality, Male"[Mesh] OR "Bisexuality"[Mesh] OR "Sexual and Gender Minorities"[Mesh] OR homosex*[tiab] OR gay[tiab] OR gays[tiab] OR bisex*[tiab] OR bi-sex*[tiab] OR msm[tiab] OR men who have sex with men[tiab] OR "Transgender Persons"[Mesh] OR transgender*[tiab] OR trans-gender*[tiab] OR transwom*[tiab] OR trans-wom*[tiab] OR transman[tiab] OR transmen[tiab] OR trans-man[tiab] OR trans-men[tiab] OR male-to-female[tiab] OR female-to-male[tiab] | **63625** |
| **#2** | "Social Media"[Mesh] OR "social media"[tiab] OR facebook[tiab] OR "face book"[tiab] OR social network*[tiab] OR social market*[tiab] OR crowdsourc*[tiab] OR crowd sourc*[tiab] OR e-learning*[tiab] OR elearning*[tiab] OR instagram*[tiab] OR myspace[tiab] OR reddit[tiab] OR tumblr[tiab] OR renren[tiab] OR grindr[tiab] OR hornet[tiab] OR scruff[tiab] OR tinder[tiab] OR “jack’d”[tiab] OR banner*[tiab] OR dating*[tiab] OR weibo[tiab] OR whatsapp*[tiab] OR wechat[tiab] OR online[tiab] OR bathhous*[tiab] OR bath hous*[tiab] OR sauna*[tiab] OR multimedia*[tiab] OR multi-media*[tiab] OR chat room*[tiab] OR chatroom*[tiab] OR internet*[tiab] OR application*[tiab] OR mobile app*[tiab] OR app[ti] OR apps[ti] OR online dating[tiab] OR “hook up app”[tiab] OR “hook up apps”[tiab] OR "Health Knowledge, Attitudes, Practice"[Mesh] OR "Patient Education as Topic"[Mesh] OR "Patient Education Handout" [Publication Type] OR "Patient Selection"[Mesh] OR risk scor* OR riskscor* OR risk based score*[tiab] OR symptom-based score*[tiab] OR risk screening scor*[tiab] OR umrss[tiab] OR symptoms checklist*[tiab] OR symptom checklist*[tiab] OR educat*[tiab] OR communicat*[tiab] OR campaign*[tiab] OR mobilis*[tiab] OR mobiliz*[tiab] OR recruit*[tiab] OR target*[tiab] OR understand*[tiab] OR “self-referral test”[tiab] OR early test[tiab] OR self test*[tiab] OR "Knowledge"[Mesh] OR knowledge*[tiab] OR "Communication"[Mesh] OR "education" [Subheading] OR training*[tiab] | **5734612** |
| **#1** | (("HIV"[Mesh] OR human immunodeficiency virus*[ti] OR hiv*[ti]) AND (acute[ti] OR early[ti] OR primary[ti] OR recent*[ti])) OR acute human immunodeficiency virus*[tiab] OR early human immunodeficiency virus*[tiab] OR primary human immunodeficiency virus*[tiab] OR recent human immunodeficiency virus*[tiab] OR acute hiv*[tiab] OR early hiv*[tiab] OR primary hiv*[tiab] OR recent hiv*[tiab] OR recently acquired hiv*[tiab] | **12578** |

# Embase.com 25 May 2020 (897)

| **No.** | **Query** | **Items** |
| --- | --- | --- |
| **#5** | #4 NOT *[450 PMIDs]* | **515** |
| **#4** | #1 AND #2 AND #3 | **897** |
| **#3** | 'homosexuality'/de OR 'male homosexuality'/exp OR 'bisexuality'/exp OR 'sexual and gender minority'/exp OR 'transgender'/exp OR homosex*:ti,ab,kw OR gay:ti,ab,kw OR gays:ti,ab,kw OR bisex*:ti,ab,kw OR 'bi sex*':ti,ab,kw OR msm:ti,ab,kw OR 'men who have sex with men':ti,ab,kw OR transgender*:ti,ab,kw OR 'trans gender*':ti,ab,kw OR transwom*:ti,ab,kw OR 'trans wom*':ti,ab,kw OR transman:ti,ab,kw OR transmen:ti,ab,kw OR 'trans man':ti,ab,kw OR 'trans men':ti,ab,kw OR 'male to female':ti,ab,kw OR 'female to male':ti,ab,kw | **87342** |
| **#2** | 'social media'/exp OR 'social network'/exp OR 'crowdsourcing'/exp OR 'mobile application'/exp OR 'online dating'/exp OR 'support group'/exp OR 'internet'/exp OR 'sauna'/exp OR 'multimedia'/exp OR 'interpersonal communication'/exp OR 'knowledge'/de OR 'attitude to health'/exp OR 'health education'/de OR 'health promotion'/exp OR 'health literacy'/exp OR 'hiv education'/exp OR 'patient education'/exp OR 'patient selection'/exp OR 'education'/de OR 'sexual education'/exp OR 'electronic learning'/exp OR 'dating'/exp OR 'risk score'/exp OR 'social media':ti,ab,kw OR facebook:ti,ab,kw OR 'face book':ti,ab,kw OR 'social network*':ti,ab,kw OR 'social market*':ti,ab,kw OR crowdsourc*:ti,ab,kw OR 'crowd sourc*':ti,ab,kw OR 'e learning*':ti,ab,kw OR elearning*:ti,ab,kw OR instagram*:ti,ab,kw OR myspace:ti,ab,kw OR reddit:ti,ab,kw OR tumblr:ti,ab,kw OR renren:ti,ab,kw OR grindr:ti,ab,kw OR hornet:ti,ab,kw OR scruff:ti,ab,kw OR tinder:ti,ab,kw OR 'jack d':ti,ab,kw OR banner*:ti,ab,kw OR dating*:ti,ab,kw OR weibo:ti,ab,kw OR whatsapp*:ti,ab,kw OR wechat:ti,ab,kw OR online:ti,ab,kw OR bathhous*:ti,ab,kw OR 'bath hous*':ti,ab,kw OR sauna*:ti,ab,kw OR multimedia*:ti,ab,kw OR 'multi media*':ti,ab,kw OR 'chat room*':ti,ab,kw OR chatroom*:ti,ab,kw OR internet*:ti,ab,kw OR application*:ti,ab,kw OR 'mobile app*':ti,ab,kw OR app:ti,kw OR apps:ti,kw OR 'hook up app*':ti,ab,kw OR ((risk NEAR/2 scor*):ti,ab,kw) OR riskscor*:ti,ab,kw OR ((symptom NEAR/2 score*):ti,ab,kw) OR umrss:ti,ab,kw OR 'symptoms checklist*':ti,ab,kw OR 'symptom checklist*':ti,ab,kw OR educat*:ti,ab,kw OR communicat*:ti,ab,kw OR campaign*:ti,ab,kw OR mobilis*:ti,ab,kw OR mobiliz*:ti,ab,kw OR recruit*:ti,ab,kw OR target*:ti,ab,kw OR understand*:ti,ab,kw OR 'self referral test':ti,ab,kw OR 'early test*':ti,ab,kw OR 'self test*':ti,ab,kw OR knowledge*:ti,ab,kw OR training*:ti,ab,kw | **7641833** |
| **#1** | 'acute hiv infection'/exp OR (('human immunodeficiency virus'/exp OR 'human immunodeficiency virus*':ti,kw OR hiv*:ti,kw) AND (acute:ti,kw OR early:ti,kw OR primary:ti,kw OR recent*:ti,kw)) OR 'acute human immunodeficiency virus*':ti,ab,kw OR 'early human immunodeficiency virus*':ti,ab,kw OR 'primary human immunodeficiency virus*':ti,ab,kw OR 'recent human immunodeficiency virus*':ti,ab,kw OR 'acute hiv*':ti,ab,kw OR 'early hiv*':ti,ab,kw OR 'primary hiv*':ti,ab,kw OR 'recent hiv*':ti,ab,kw OR 'recently acquired hiv*':ti,ab,kw | **19764** |

# Clarivate Analytics/Web of Science Core Collection 25 May 2020 (280)

| **Set** | **Query** | **Items** |
| --- | --- | --- |
| **#5** | #4 NOT *[450 PMIDs]* | **43** |
| **# 4** | #3 AND #2 AND #1 | **280** |
| **# 3** | TOPIC: (“homosex*” OR “gay” OR “gays” OR “bisex*” OR “bi sex*” OR “msm” OR “men who have sex with men” OR “transgender*” OR “trans gender*” OR “transwom*” OR “trans wom*” OR “transman” OR “transmen” OR “trans man” OR “trans men” OR “male to female” OR “female to male”) | **84,733** |
| **# 2** | TOPIC: (“social media” OR “facebook” OR “face book” OR “social network*” OR “social market*” OR “crowdsourc*” OR “crowd sourc*” OR “e learning*” OR “elearning*” OR “instagram*” OR “myspace” OR “reddit” OR “tumblr” OR “renren” OR “grindr” OR “hornet” OR “scruff” OR “tinder” OR “jack d” OR “banner*” OR “dating*” OR “weibo” OR “whatsapp*” OR “wechat” OR “online” OR “bathhous*” OR “bath hous*” OR “sauna*” OR “multimedia*” OR “multi media*” OR “chat room*” OR “chatroom*” OR “internet*” OR “application*” OR “mobile app*” OR “app” OR “apps” OR “hook up app*” OR (“risk” NEAR/2 “scor*”) OR “riskscor*” OR (“symptom” NEAR/2 “score*”) OR “umrss” OR “symptoms checklist*” OR “symptom checklist*” OR “educat*” OR “communicat*” OR “campaign*” OR “mobilis*” OR “mobiliz*” OR “recruit*” OR “target*” OR “understand*” OR “self referral test” OR “early test*” OR “self test*” OR “knowledge*” OR “training*”) | **9,598,579** |
| **# 1** | TOPIC: (“acute human immunodeficiency virus*” OR “early human immunodeficiency virus*” OR “primary human immunodeficiency virus*” OR “recent human immunodeficiency virus*” OR “acute hiv*” OR “early hiv*” OR “primary hiv*” OR “recent hiv*” OR “recently acquired hiv*”) | **5,769** |

# Ebsco/ERIC 25 May 2020 (5)

| **#** | **Query** | **Items** |
| --- | --- | --- |
| **S4** | S1 AND S2 AND S3 | **5** |
| **S3** | ( DE "Acquired Immunodeficiency Syndrome (AIDS)" OR TI(“human immunodeficiency virus*” OR hiv*) OR KW((human W0 immunodeficiency W0 virus*) OR hiv*) ) AND ( TI(acute OR early OR primary OR recent*) OR KW(acute OR early OR primary OR recent*) ) OR TI(“acute human immunodeficiency virus*” OR “early human immunodeficiency virus*” OR “primary human immunodeficiency virus*” OR “recent human immunodeficiency virus*” OR “acute hiv*” OR “early hiv*” OR “primary hiv*” OR “recent hiv*” OR “recently acquired hiv*”) OR AB(“acute human immunodeficiency virus*” OR “early human immunodeficiency virus*” OR “primary human immunodeficiency virus*” OR “recent human immunodeficiency virus*” OR “acute hiv*” OR “early hiv*” OR “primary hiv*” OR “recent hiv*” OR “recently acquired hiv*”) OR KW(((acute OR early OR primary OR recent) N3 (human W0 immunodeficiency W0 virus*)) OR (acute W0 hiv*) OR (early W0 hiv*) OR (primary W0 hiv*) OR (recent W0 hiv*) OR (recently W0 acquired W0 hiv*)) | **20,148** |
| **S2** | DE "Access to Information" OR DE "Adult Education" OR DE "Adult Learning" OR DE "Audiovisual Aids" OR DE "Audiovisual Communications" OR DE "Audiovisual Instruction" OR DE "Classroom Research" OR DE "Communication (Thought Transfer)" OR DE "Communications" OR DE "Community Education" OR DE "Community Information Services" OR DE "Comprehensive School Health Education" OR DE "Computer Assisted Instruction" OR DE "Computer Assisted Testing" OR DE "Computer Games" OR DE "Computer Managed Instruction" OR DE "Computer Mediated Communication" OR DE "Computer Oriented Programs" OR DE "Computer Software" OR DE "Computer Use" OR DE "Computer Uses in Education" OR DE "Courseware" OR DE "Curriculum Research" OR DE "Database Management Systems" OR DE "Dating (Social)" OR DE "Educational Games" OR DE "Educational Needs" OR DE "Educational Objectives" OR DE "Educational Practices" OR DE "Educational Research" OR DE "Educational Strategies" OR DE "Educational Television" OR DE "Effective Schools Research" OR DE "Electronic Learning" OR DE "Electronic Mail" OR DE "Expertise" OR DE "Games" OR DE "General Education" OR DE "Global Education" OR DE "Handheld Devices" OR DE "Health Education" OR DE "Health Promotion" OR DE "Informal Education" OR DE "Information Management" OR DE "Information Networks" OR DE "Information Services" OR DE "Information Skills" OR DE "Information Transfer" OR DE "Instructional Films" OR DE "Integrated Learning Systems" OR DE "Interactive Video" OR DE "Internet" OR DE "Interpersonal Communication" OR DE "Knowledge Level" OR DE "Laptop Computers" OR DE "Learning Motivation" OR DE "Learning" OR DE "Mass Media" OR DE "Multimedia Instruction" OR DE "Multimedia Materials" OR DE "Nonformal Education" OR DE "Online Courses" OR DE "Patient Education" OR DE "Public Education" OR DE "Public School Adult Education" OR DE "Reading Research" OR DE "Safety Education" OR DE "Search Engines" OR DE "Sex Education" OR DE "Social Media" OR DE "Social Networks" OR DE "Social Support Groups" OR DE "Speech Communication" OR DE "Telecommunications" OR DE "Television" OR DE "Training" OR DE "Urban Education" OR DE "Users (Information)" OR DE "Verbal Communication" OR DE "Verbal Learning" OR DE "Video Games" OR DE "Video Technology" OR DE "Virtual Classrooms" OR DE "Visual Aids" OR DE "Web 2.0 Technologies" OR DE "Web Based Instruction" OR DE "Web Browsers" OR DE "Web Sites" OR DE "Workstations" OR TI(“social media” OR facebook OR “face book” OR “social network*” OR “social market*” OR crowdsourc* OR “crowd sourc*” OR “e learning*” OR elearning* OR instagram* OR myspace OR reddit OR tumblr OR renren OR grindr OR hornet OR scruff OR tinder OR “jack d” OR banner* OR dating* OR weibo OR whatsapp* OR wechat OR online OR bathhous* OR “bath hous*” OR sauna* OR multimedia* OR “multi media*” OR “chat room*” OR chatroom* OR internet* OR application* OR “mobile app*” OR app:ti,kw OR apps:ti,kw OR “hook up app*” OR (risk N2 scor*) OR riskscor* OR (symptom N2 score*) OR umrss OR “symptoms checklist*” OR “symptom checklist*” OR educat* OR communicat* OR campaign* OR mobilis* OR mobiliz* OR recruit* OR target* OR understand* OR “self referral test” OR “early test*” OR “self test*” OR knowledge* OR training*) OR AB(“social media” OR facebook OR “face book” OR “social network*” OR “social market*” OR crowdsourc* OR “crowd sourc*” OR “e learning*” OR elearning* OR instagram* OR myspace OR reddit OR tumblr OR renren OR grindr OR hornet OR scruff OR tinder OR “jack d” OR banner* OR dating* OR weibo OR whatsapp* OR wechat OR online OR bathhous* OR “bath hous*” OR sauna* OR multimedia* OR “multi media*” OR “chat room*” OR chatroom* OR internet* OR application* OR “mobile app*” OR app:ti,kw OR apps:ti,kw OR “hook up app*” OR (risk N2 scor*) OR riskscor* OR (symptom N2 score*) OR umrss OR “symptoms checklist*” OR “symptom checklist*” OR educat* OR communicat* OR campaign* OR mobilis* OR mobiliz* OR recruit* OR target* OR understand* OR “self referral test” OR “early test*” OR “self test*” OR knowledge* OR training*) OR KW((social W0 media) OR facebook OR (face W0 book) OR (social W0 network*) OR (social W0 market*) OR crowdsourc* OR (crowd W0 sourc*) OR (e W0 learning*) OR elearning* OR instagram* OR myspace OR reddit OR tumblr OR renren OR grindr OR hornet OR scruff OR tinder OR (jack W0 d) OR banner* OR dating* OR weibo OR whatsapp* OR wechat OR online OR bathhous* OR (bath W0 hous*) OR sauna* OR multimedia* OR (multi W0 media*) OR (chat W0 room*) OR chatroom* OR internet* OR application* OR (mobile W0 app*) OR app:ti,kw OR apps:ti,kw OR (hook W0 up W0 app*) OR (risk N2 scor*) OR riskscor* OR (symptom N2 score*) OR umrss OR (symptoms W0 checklist*) OR (symptom W0 checklist*) OR educat* OR communicat* OR campaign* OR mobilis* OR mobiliz* OR recruit* OR target* OR understand* OR (self W0 referral W0 test) OR (early W0 test*) OR (self W0 test*) OR knowledge* OR training*) | **1,187,694** |
| **S1** | ( DE "Acquired Immunodeficiency Syndrome (AIDS)" OR TI(“human immunodeficiency virus*” OR hiv*) OR KW((human W0 immunodeficiency W0 virus*) OR hiv*) ) AND ( TI(acute OR early OR primary OR recent*) OR KW(acute OR early OR primary OR recent*) ) OR TI(“acute human immunodeficiency virus*” OR “early human immunodeficiency virus*” OR “primary human immunodeficiency virus*” OR “recent human immunodeficiency virus*” OR “acute hiv*” OR “early hiv*” OR “primary hiv*” OR “recent hiv*” OR “recently acquired hiv*”) OR AB(“acute human immunodeficiency virus*” OR “early human immunodeficiency virus*” OR “primary human immunodeficiency virus*” OR “recent human immunodeficiency virus*” OR “acute hiv*” OR “early hiv*” OR “primary hiv*” OR “recent hiv*” OR “recently acquired hiv*”) OR KW(((acute OR early OR primary OR recent) N3 (human W0 immunodeficiency W0 virus*)) OR (acute W0 hiv*) OR (early W0 hiv*) OR (primary W0 hiv*) OR (recent W0 hiv*) OR (recently W0 acquired W0 hiv*)) | **78** |

Table S2. The Appraisal tool for Cross-Sectional Studies (AXIS)

|  | | **Yes** | **No** | **Don’t know / comment** |
| --- | --- | --- | --- | --- |
| **Introduction** | |  |  |  |
| 1 | Were the aims/objectives of the study clear? |  |  |  |
| **Methods** | |  |  |  |
| 2 | Was the study design appropriate for the stated aim(s)? |  |  |  |
| 3 | Was the sample size justified? |  |  |  |
| 4 | Was the target/reference population clearly defined? (Is it clear who the research was about?) |  |  |  |
| 5 | Was the sample frame taken from an appropriate population base so that it closely represented the target/reference population under investigation? |  |  |  |
| 6 | Was the selection process likely to select subjects/participants that were representative of the target/reference population under investigation? |  |  |  |
| 7 | Were measures undertaken to address and categorise non-responders? |  |  |  |
| 8 | Were the risk factor and outcome variables measured appropriate to the aims of the study? |  |  |  |
| 9 | Were the risk factor and outcome variables measured correctly using instruments/measurements that had been trialled, piloted or published previously? |  |  |  |
| 10 | Is it clear what was used to determined statistical significance and/or precision estimates? (e.g. p-values, confidence intervals) |  |  |  |
| 11 | Were the methods (including statistical methods) sufficiently described to enable them to be repeated? |  |  |  |
| **Results** | |  |  |  |
| 12 | Were the basic data adequately described? |  |  |  |
| 13 | Does the response rate raise concerns about non-response bias? |  |  |  |
| 14 | If appropriate, was information about non-responders described? |  |  |  |
| 15 | Were the results internally consistent? |  |  |  |
| 16 | Were the results presented for all the analyses described in the methods? |  |  |  |
| **Discussion** | |  |  |  |
| 17 | Were the authors' discussions and conclusions justified by the results? |  |  |  |
| 18 | Were the limitations of the study discussed? |  |  |  |
| **Other** | |  |  |  |
| 19 | Were there any funding sources or conflicts of interest that may affect the authors’ interpretation of the results? |  |  |  |
| 20 | Was ethical approval or consent of participants attained? |  |  |  |

Table S3. Critical appraisal of included studies by using the Appraisal Tool for Cross-Sectional Studies

|  | **Question number** | | | | | | | | | | | | | | | | | | | | |
| --- | --- | --- | --- | --- | --- | --- | --- | --- | --- | --- | --- | --- | --- | --- | --- | --- | --- | --- | --- | --- | --- |
|  | **1.** | **2.** | **3.** | **4.** | **5.** | **6.** | **7.** | **8.** | **9.** | | **10.** | **11.** | **12.** | **13.** | **14.** | **15.** | **16.** | **17.** | **18.** | **19.** | **20.** |
| Required answer for good quality | Y | Y | Y | Y | Y | Y | Y | Y | Y | | Y | Y | Y | N | Y | Y | Y | Y | Y | N | Y |
| **Studies** | | | | | | | | | | | | | | | | | | | | | |
| Mobilisation strategies | | | | | | | | | | | | | | | | | | | | | |
| Silvera (2010) | Y | Y | N | Y | Y | Y | N | Y | Y | | N | Y | Y | D/K | N | Y | Y | Y | N | N | Y |
| Stekler  (2013) | Y | Y | N | Y | Y | Y | N | Y | Y | | N | N | Y | N | N | Y | Y | Y | N | N | Y |
| Gilbert  (2013) | Y | Y | N | Y | Y | Y | N | Y | Y | | Y | Y | Y | N | N | Y | Y | Y | N | N | Y |
| Dijkstra  (2020) | Y | Y | N | Y | Y | Y | Y | Y | Y | | Y | Y | Y | Y | Y | Y | Y | Y | Y | N | Y |
| Green  (2017) | Y | Y | N | Y | Y | Y | Y | Y | Y | | Y | Y | Y | Y | Y | Y | Y | Y | Y | N | Y |
| Daskalakis  (2009) | Y | Y | N | Y | Y | Y | Y | Y | Y | | Y | Y | Y | Y | N | Y | Y | Y | Y | N | Y |
| Liang  (2015) | Y | Y | N | Y | Y | Y | N | Y | Y | | Y | Y | Y | Y | Y | Y | Y | Y | Y | N | Y |
| Pankam  (2018) | Y | Y | N | Y | Y | Y | N | Y | Y | | Y | Y | Y | D/K | N | Y | Y | Y | N | D/K | Y |
| Risk and/or symptom scores | | | | | | | | | | | | | | | | | | | | | |
| Menza (2009) | Y | Y | N | Y | Y | Y | N | Y | Y | Y | | Y | Y | Y | Y | Y | Y | Y | Y | D/K | Y |
| Facente (2011) | Y | Y | N | Y | Y | Y | Y | Y | Y | Y | | Y | Y | N | Y | Y | Y | Y | Y | N | Y |
| Smith (2012) | N | Y | N | Y | D/K | N | D/K | Y | Y | Y | | Y | Y | D/K | Y | Y | Y | Y | Y | N | D/K |
| Wahome (2013) | Y | Y | N | Y | D/K | D/K | N | Y | Y | Y | | Y | Y | D/K | N | Y | Y | Y | Y | N | D/K |
| Hoenigl (2015) | Y | Y | N | Y | Y | Y | Y | Y | Y | Y | | Y | Y | N | Y | Y | Y | Y | Y | N | Y |
| Sanders (2015) | Y | Y | N | Y | D/K | D/K | N | Y | Y | Y | | Y | Y | D/K | N | Y | Y | Y | Y | N | D/K |
| Beymer (2017) | Y | Y | N | Y | Y | Y | Y | Y | Y | Y | | Y | Y | D/K | N | Y | Y | Y | Y | N | Y |
| Jones (2017) | Y | Y | N | Y | Y | Y | D/K | Y | Y | Y | | Y | Y | D/K | N | Y | Y | Y | Y | N | D/K |
| Dijkstra (2017) | Y | Y | N | Y | Y | N | D/K | Y | Y | Y | | Y | Y | D/K | N | Y | Y | Y | Y | N | Y |
| Lancki (2018) | Y | Y | N | Y | Y | Y | Y | Y | Y | Y | | Y | Y | N | Y | Y | Y | Y | Y | N | Y |
| Wahome (2018) | Y | Y | N | Y | Y | Y | N | Y | Y | Y | | Y | Y | D/K | N | Y | Y | Y | Y | N | Y |
| Lin  (2018) | Y | Y | N | Y | Y | Y | N | Y | Y | Y | | Y | Y | N | Y | Y | Y | Y | Y | N | Y |
| Lin  (2018) | Y | Y | N | Y | Y | Y | N | Y | Y | Y | | Y | Y | D/K | N | Y | Y | Y | Y | N | Y |
| Dijkstra (2019) | Y | Y | N | Y | D/K | D/K | N | Y | Y | Y | | Y | Y | D/K | N | Y | Y | Y | Y | N | Y |

D/K, don’t know; N, no; Y, yes. The black colours represent ‘good quality’, the red colours represent ‘poor quality’ on the specified item of the appraisal tool. Don’t know was used if the study did not report on the specified item.
